# Operational complexity versus design efficiency: challenges of implementing a phase IIa multiple parallel cohort targeted treatment platform trial in advanced breast cancer

**DOI:** 10.1186/s13063-022-06312-x

**Published:** 2022-05-07

**Authors:** Claire Snowdon, Sarah Kernaghan, Laura Moretti, Nicholas C. Turner, Alistair Ring, Katie Wilkinson, Sue Martin, Stephanie Foster, Lucy S. Kilburn, Judith M. Bliss

**Affiliations:** 1 Clinical Trials and Statistics Unit, The Institute of Cancer Research, London, UK; 2 The Institute of Cancer Research Clinical Trials and Statistics Unit, 15 Cotswold Road, Sutton, Surrey SM2 5NG UK; 3 Breast Cancer Now Research Centre, The Institute of Cancer Research, London, UK; 4 Breast Unit, The Royal Marsden Hospital NHS Foundation Trust, London, UK

**Keywords:** Platform trial, Complex design, Efficient, Operational challenges

## Abstract

**Background:**

Platform trial designs are used increasingly in cancer clinical research and are considered an efficient model for evaluating multiple compounds within a single disease or disease subtype. However, these trial designs can be challenging to operationalise.

The use of platform trials in oncology clinical research has increased considerably in recent years as advances in molecular biology enable molecularly defined stratification of patient populations and targeted therapy evaluation. Whereas multiple separate trials may be deemed infeasible, platform designs allow efficient, parallel evaluation of multiple targeted therapies in relatively small biologically defined patient sub-populations with the promise of increased molecular screening efficiency and reduced time for drug evaluation. Whilst the theoretical efficiencies are widely reported, the operational challenges associated with these designs (complexity, cost, regulatory, resource) are not always well understood.

**Main:**

In this commentary, we describe our practical experience of the implementation and delivery of the UK plasmaMATCH trial, a platform trial in advanced breast cancer, comprising an integrated screening component and multiple parallel downstream mutation-directed therapeutic cohorts. plasmaMATCH reported its primary results within 3 years of opening to recruitment. We reflect on the operational challenges encountered and share lessons learnt to inform the successful conduct of future trials. Key to the success of the plasmaMATCH trial was well co-ordinated stakeholder engagement by an experienced clinical trials unit with expert methodology and trial management expertise, a federated model of clinical leadership, a well-written protocol integrating screening and treatment components and including justification for the chosen structure and intentions for future adaptions, and an integrated funding model with streamlined contractual arrangements across multiple partners. Findings based on our practical experience include the importance of early engagement with the regulators and consideration of a flexible resource infrastructure to allow adequate resource allocation to support concurrent trial activities as adaptions are implemented in parallel to the continued management of patient safety and data quality of the ongoing trial cohorts.

**Conclusion:**

Platform trial designs allow the efficient reporting of multiple treatment cohorts. Operational challenges can be overcome through multidisciplinary engagement, streamlined contracting processes, rationalised protocol and database design and appropriate resourcing.

## Background

Platform trials allow the evaluation of several interventions within stratified patient sub-populations under a single trial with the opportunity for further additions or exclusions of new therapies or biomarkers to be made in response to accumulating data within the trial [1–4]. Thus creating a trial platform which brings together a suite of studies that would have historically been conducted separately enables the study of treatment effects in multiple and small patient sub-populations to be feasible in practice.

The envisaged efficiencies of the use of platform trials, based on a theoretical framework, are well recognised, including increased screening efficiency and reduced time for drug evaluation [5–9]. For this reason, platform trials were widely used during the covid-19 pandemic as an efficient means to investigate a new disease and multiple treatments which became available at the same time across a very large patient population.

In recent years, there has been a notable increase in the use of platform trial designs in oncology clinical research [3, 9, 10] reflecting the advancement of personalised medicine initiatives in response to a greater understanding of the heterogeneity of cancer. The identification of specific genetic mutations and biological categorisation of further patient sub-populations, often occurring at a low frequency, along with the development of associated targeted therapies presents the opportunity for clinical trial designs to adapt in response [4, 5, 7, 11–13]. Biomarker-guided trials with molecular screening (often next generation sequencing (NGS)) provide the opportunity to identify patient sub-populations most likely to benefit from a given treatment and are pivotal in the development of personalised medicine, but can present challenges in practice [14].

Whilst the scientific efficiencies associated with the use of platform trials are well recognised, the operational challenges, which are multiple, are less well understood. Here we discuss in detail these challenges using the UK plasmaMATCH trial as a practical example of implementation and delivery of a platform trial with an integrated molecular screening component. Our key recommendations based on our practical experience are described in Table 1 at the end of this commentary.

**Table 1:**
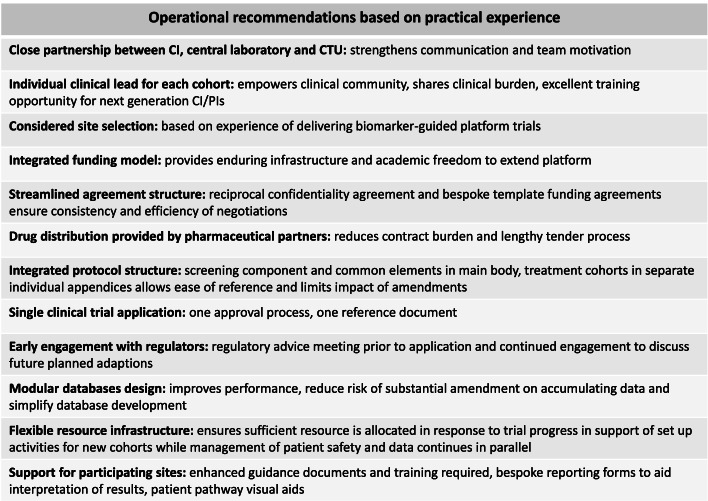
Operational recommendations based on practical experience

## Main

### plasmaMATCH trial design and clinical results

**Fig. 1:**
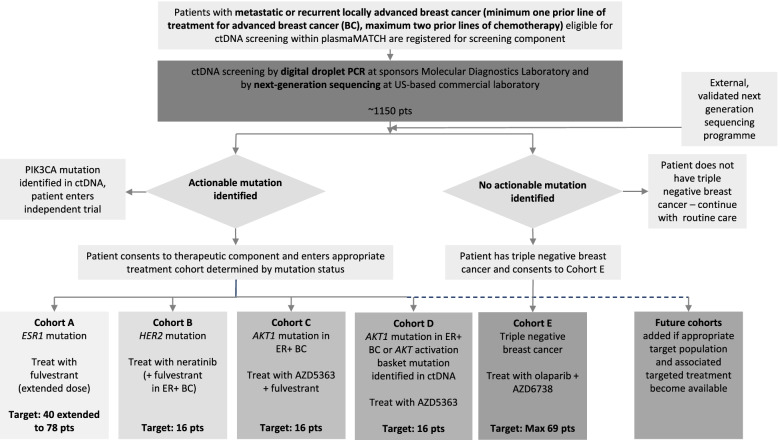
plasmaMATCH trial design

The UK-based plasmaMATCH trial [15] [ISRCTN16945804, NCT03182634, CRUK/15/010] (Fig. 1, plasmaMATCH trial design) is a multiple-parallel cohort, open-label, multi-centre phase IIa platform clinical trial with an integrated molecular screening component aiming to provide proof of principle activity for designated targeted therapies in patients with advanced breast cancer. The trial is co-sponsored by The Institute of Cancer Research (ICR) and The Royal Marsden NHS Foundation Trust (RMH). Its novelty relates to the identification of a targetable mutation from circulating tumour DNA (ctDNA) utilising liquid biopsy-based screening to direct entry into a therapeutic cohort. Eligible patients with advanced breast cancer were registered for ctDNA screening by droplet digital PCR at the co-sponsor’s central laboratory. Patients with an actionable mutation were invited to consent to a corresponding treatment cohort. Mutations identified via an external validated sequencing programme were also eligible for entry into the treatment component.

The original design consisted of four treatment cohorts, A-D, with a target accrual of 40 patients in cohort A and 16 patients in each of cohorts B–D requiring >1000 patients to be registered for ctDNA screening to identify the target number of patients to enter the treatment cohorts due to the rarity of the actionable mutations. The trial was designed with the intention that modifications would be made in response to emerging trial data and future cohorts could be added if relevant matched populations and associated targeted treatments became available. As such, cohort A was extended from 40 to 78 patients due to the emerging evidence of sub-clonal mutational frequency which led to higher overall mutational frequency than originally predicted and cohort E (target accrual 69 patients maximum) was added enabling patients with triple-negative breast cancer (TNBC) but no actionable mutation identified to enter a therapeutic cohort (biomarker exclusion cohort). A second method for mutation analysis by NGS at a US-based laboratory was introduced part way through the trial. The trial design, including adaptions, is shown in Fig. 1, plasmaMATCH trial design.

The main clinical results for the screening component and initial four treatment cohorts A–D were reported within 3 years of opening to recruitment [15] and the trial successfully identified two mutations with clinically relevant activity observed following treatment with paired targeted therapies. The results for cohort E will also be reported within 3.5 years of opening that cohort to recruitment despite the impact of the covid-19 pandemic.

### Multidisciplinary engagement

Clinical trials require a collaborative effort involving multidisciplinary engagement; the more complex the design, the greater the number of stakeholders involved. This is particularly evident for biomarker-driven platform designs that often rely on increased clinical and methodology input including early engagement with oversight committees, trial management expertise and co-operation between numerous specialist research laboratories and support from multiple pharmaceutical partners and funders [5, 8]. This can add significant logistical complexity.

**Fig. 2:**
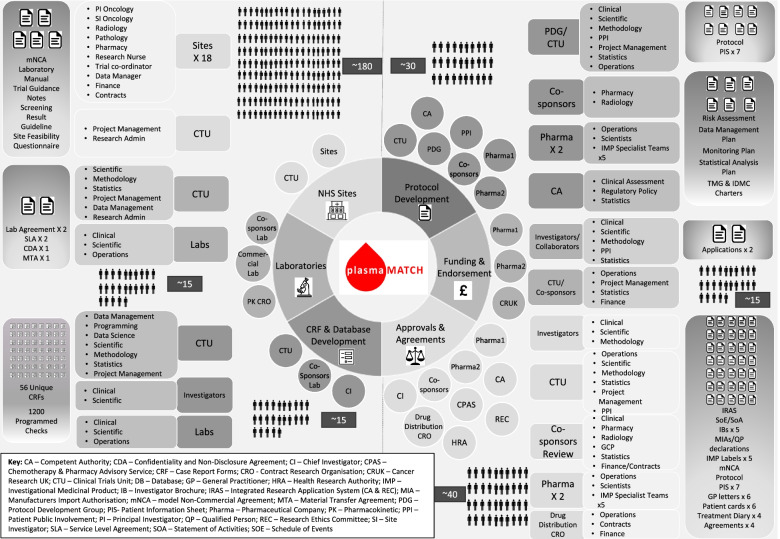
Multidisciplinary engagement illustrated by plasmaMATCH shows a visual representation of the extensive collaborative nature of a platform trial using plasmaMATCH as an example

Experienced clinical trials units (CTUs) have the quality systems; expert staff, including academic trial methodologists; and operational project management infrastructure vital to manage the multiple stakeholders and complex logistics to optimise platform trial delivery on behalf of the sponsor. The ICR Clinical Trials and Statistics Unit (ICR-CTSU), an academic CTU based in London, co-ordinated the plasmaMATCH trial and was responsible for the statistical design and analysis. The CTU team, consisting of trial management, data management, statistical, programming and administration staff, were experienced in the management and analysis of multi-centre, bio-sample rich, targeted treatment trials in breast cancer and acted as the *glue* in the complex trial collaboration. Trial initiation and conduct required co-ordination of a team of multidisciplinary experts from approximately 30 academic, hospital and commercial organisations (not including regulatory and other approval bodies and oversight committees) (Fig. 2, multidisciplinary engagement illustrated by plasmaMATCH).

Clinical leadership was shared between five nationally located co-investigators, each named as a specific treatment cohort clinical lead within the protocol. This allowed the increased clinical burden associated with a platform trial (multiple unlicensed/novel investigational medicinal products (IMPs) requiring continued assessment of risk-benefit ratio with accumulation of external evidence, increased pharmacovigilance activities and development and regular review of the high level of trial documentation (protocol, multiple patient information sheets (PIS), informed consent forms (ICFs), general practitioner (GP) letters) to be shared. The benefit of this federated approach also enhanced national distribution of clinical input and trial oversight, strengthening collaboration and investigator buy-in, offsets the potential for portfolio dominance by a single investigator and provides excellent training opportunities for the next generation of chief investigators (CIs).

Mutational screening and other trial-specific biomarker research within plasmaMATCH involved six laboratories, both national and international. Close collaboration between the CTU and the specialist research laboratories involved in a biomarker directed platform trial is vital for the co-ordination of sample receipt and achievement of rapid turnaround times for mutational screening results.

Platform trials, such as plasmaMATCH, that are designed to identify biologically driven patient sub-populations often by identification of rare mutations require national collaboration to achieve target recruitment. Eighteen participating sites spread across the UK, selected based on their experience of delivering biomarker-guided trials in advanced breast cancer, contributed patients to plasmaMATCH. Sites were required to identify and name the local multidisciplinary team (oncologist, radiologist, pathologist, research nurse) responsible for the delivery of the trial. This ensured buy-in from all relevant departments during trial initiation and contributed to higher than anticipated recruitment rate from the outset.

### Trial funding and agreements

Quantification of the future scale and complexity of a dynamic platform trial is difficult to define at the outset making costing these designs challenging [14]. Due to their inherent size and complexity, the costs associated with delivering platforms can be high [2, 7] and traditional costing approaches do not have the requisite flexibility to accommodate such aspects. Funders may be unable or unwilling to fund them in their entirety due to the price and potential for additional undefined associated costs. An integrated funding model with multiple funding partners necessarily leads to complex budget and contract negotiations [2, 7] including intellectual property attribution. In plasmaMATCH, Cancer Research UK, the largest non-commercial funder of academic cancer trials in the UK, funded the screening platform and overarching trial infrastructure costs, whilst individual treatment cohorts were funded by research grants from the corresponding pharmaceutical partners. The co-sponsor’s National Institute for Health Research (NIHR) Biomedical Research Centre funding supported elements of additional translational research, including on and end of treatment ctDNA analysis. This integrated funding model was intentional and was important to provide an enduring infrastructure and to protect academic freedom to extend the platform.

To streamline the agreement structure, and to future-proof for the addition of potential new treatment cohorts, a reciprocal confidentiality agreement was implemented between the co-sponsors and pharmaceutical partners to allow further companies to join later and to overcome the added complication of combination treatment cohorts involving more than one pharmaceutical partner. A bespoke template funding agreement was developed and required all current, and any future, pharmaceutical partners to sign up to the same terms and conditions and allowed efficient amendment for future cohort adaptations or additions. This model also worked to the advantage of the academic co-sponsors with increased bargaining power at contract negotiation in order to ensure terms were kept consistent across pharmaceutical partners. Pharmaceutical partners were also required to provide drug distribution thereby reducing the co-sponsor’s agreement burden further and eliminating the need for time consuming drug distribution vendor selection processes. The agreement structure resulted in reduced and simplified contractual negotiations which in turn avoided unnecessary delay at trial set-up.

### Regulatory

The regulatory challenge faced by platform designs results from the current lack of regulatory framework for a dynamic evolving trial design particularly in terms of the definition of end of trial for reporting purposes [1, 16]. A number of regulatory guidance documents focusing on the conduct of clinical trials utilising master protocols and adaptive design were in development at the time of writing this commentary [17, 18] and highlight concerns regarding data transparency and integrity. The European regulatory framework as implemented does not support the concept of multiple parallel treatment cohorts completing and reporting at different stages during a trial submitted under a single clinical trial application (CTA), nor does it allow new cohorts to be added ad infinitum and left open ended in the protocol (i.e. there has to be a defined end of trial). We welcome the current opportunity for engagement and collaboration with the regulators as new regulatory guidance is developed in an attempt to keep up with the changing landscape of complex clinical trial designs.

During implementation of the plasmaMATCH trial, feedback received from the regulators made it clear that regardless of the selected protocol structure or CTA model (single protocol with sub-protocols submitted under the same CTA versus multiple protocols submitted under multiple CTAs), the key to achieving successful and efficient regulatory approval is a well written protocol. The protocol needs to clearly define specific intentions for any planned future adaptions envisaged under the master protocol and layout a detailed publication plan for the individual cohorts as they complete to alleviate regulatory concerns regarding timely reporting of results and data transparency.

**Fig. 3:**
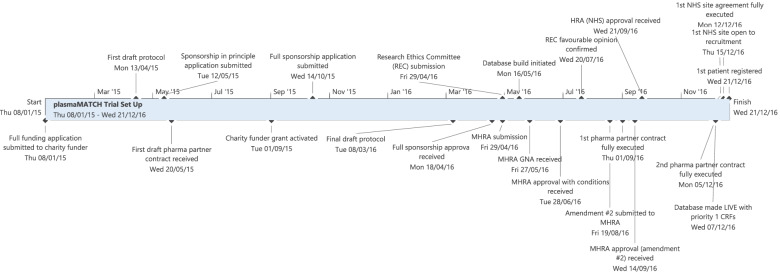
plasmaMATCH study set-up timeline

Implementing novel biomarker science, often integral to a platform trial design, can also be a regulatory challenge. A pre-submission dialogue with the regulators was not requested for plasmaMATCH as a consequence of this omission regulatory submission to approval took 5 months (Fig. 3, plasmaMATCH study set-up timeline). Direct communications with the regulator’s medical assessor during this time were found to be invaluable and helped improve mutual understanding.

Engagement with the regulatory agency’s medical assessor prior to submission of the protocol amendment for the addition of cohort E ensured an efficient approval pathway despite the challenge of adding a treatment cohort for a patient sub-population defined by lack of mutation in converse to the presence of mutation as for cohorts A–D. Based on this experience, we strongly recommend early liaison with the regulators to address any potential issues and manage the expectations of both regulators and trialists from the outset.

### Protocol structure

A key consideration when implementing a platform trial is whether a modular or integrated approach to protocol design is best [16], taking into account impact on trial set-up times (one protocol approval versus many), regulatory concerns as described previously, complexity versus performance and burden on participating sites. With a modular approach, separate protocols exist for the screening component and each individual treatment cohort allowing the timely regulatory reporting of individual cohorts under separate CTAs and reducing the risk of disclosing confidential or commercially sensitive information between multiple pharmaceutical partners. The main issues with this approach include the potential to complete the screening component before sufficient treatment cohorts have been implemented, the amount of time it takes to obtain the regulatory approvals with sequential applications for the individual elements and the administrative burden for the CTU and at participating sites in processing multiple applications. An integrated approach reduces the regulatory application and administrative burden but addition of new cohorts through protocol amendments can be logistically challenging [1, 16].

In plasmaMATCH, a single protocol integrating the screening component and individual therapeutic cohorts was implemented. The main body of the protocol described the screening and common trial elements (e.g. trial registration, pharmacovigilance, statistical considerations, trial management, oversight and governance) with therapeutic cohorts, including any potentially sensitive commercial information, described in specific separate appendices to allow ease of reference for the trial staff at participating sites and redaction when sharing the protocol with pharmaceutical partners. As a result adding in a new therapeutic cohort via amendment had limited impact on the main body of the protocol. Having one protocol meant there was only one CTA to obtain and maintain and one document for participating sites to reference. It also meant the screening and therapeutic components opened at the same time under one combined trial approval. This benefited the conversion rate from screening to cohort entry which was not subject to delay whilst approval was sought for a separate therapeutic cohort protocols. This model worked well for plasmaMATCH where sites participated in all therapeutic cohorts but may require consideration in a platform trial where sites may not be participating in all cohorts and where not all amendments apply to all sites.

Separate PIS, ICFs and GP letters were developed for each protocol element with a two-stage consent process used. Eligible patients were asked to consent to the screening component first followed by subsequent consent for treatment cohort entry if actionable mutations were identified at screening. This enabled discussions with potential patients to be focussed on detailed information relevant to each stage and treatment cohort and unavoidably resulted in greater documentation burden to be maintained by the CTU and participating sites.

### Database design

An integrated approach to database build means that participating sites have only one database to access rather than multiple separate databases; however, very large databases can be slow and unwieldy.

In plasmaMATCH, which utilised Infermed’s MACRO ™ Version 4 for electronic data capture, we opted for a single database combining the screening component with cohorts A–D to reduce the burden on participating sites. For cohort E (added later by amendment), we built a separate database due to performance issues associated with the increasing database size and concern regarding the potential risk of making significant structural changes to a database which already contained a large amount of clinical data. Although differences in visit schedules across therapeutic cohorts were kept to a minimum with the aim of consistent and simplified database development and testing, each cohort did have slightly different requirements (such as those related to treatment management) and each of these differences added to the programming complexity.

In our experience, having a separate database for cohort E did not cause a problem for participating sites and avoided performance issues associated with a single database. On reflection, separate databases for the screening component and treatment component may have improved performance. Consistency across all databases throughout the trial life cycle is key; avoiding temptation to make unnecessary ‘improvements’ or adjustments as more databases are added will avoid difficulties in the long run.

### Central trial management resource

Biologically driven, targeted treatment platform trials are challenging for trials’ staff, both within the CTU and at participating sites, due to their inherent technical complexity, the burden of documentation associated with multiple treatment cohorts [1] and the number of stakeholders involved in their delivery [5, 8]. An established centralised trials infrastructure, with appropriate quality systems and processes, is paramount [5, 14]. The complexity of these designs (both technical and logistical) can result in lengthy trial set-up times, involving multiple stakeholders, and often requiring the implementation of new systems, processes and training [7, 9], which can be at odds with funders and government expectations of trial set-up (within 1 year of funding approval in the UK). Whilst trial tasks usually happen sequentially in a traditional trial design, within platform designs, trial management and data management tasks and statistical analyses happen concurrently and continuously as new cohorts are costed and opened or closed and reported during the conduct of the trial. It is important to recognise that combining multiple sub-studies or treatment cohorts into a single protocol does not necessarily lessen the resource requirements for each individual component.

In plasmaMATCH, we opted for a staged database release, whereby priority electronic case report forms (eCRFs), including for screening and cohort entry, were released first to avoid unnecessary delay to opening the screening component of the trial. However, faster than anticipated recruitment rates and the identification of patients with actionable mutations, whilst a key success of the trial, impacted significantly on available resource, particularly in terms of further systems development due to the conflicting demands on data management and programming time associated with the staged database release coupled with high data return rates. This limited our ability to develop automated systems for data monitoring and feedback on data quality to sites in real time. A comprehensive data management plan complemented by cohort-specific project management plans was implemented to manage data cleaning and querying processes. However, sustained high recruitment rates led to challenges in keeping on top of accumulating data whilst managing the demands of adapting the platform. Three major amendments relating to platform adaptions described previously as well as multiple other trial amendments including frequent investigator brochure updates due to the use of multiple novel and unlicensed IMPs had ramifications across multiple documents and systems. New processes and training were required to support the delivery of the major trial adaptations which had resource implications. Central management of the high volume of biological trial samples, including liaison with the central laboratories and time critical reporting of screening results back to participating sites, was also resource intensive. Cleaning and analysis of cohorts in preparation for regular independent data monitoring committee (IDMC) review of emerging data (quarterly in the first 2 years) and presentation of results within the ongoing trial was challenging for data management and statistical staff, due to multiple activities happening in parallel across multiple cohorts with a high volume of accumulating data, resulting in the need to source increased data management resource from outside of the trial budget.

In plasmaMATCH, there were essentially five trials and a screening platform running under the umbrella of one protocol. More resource was budgeted for than for a single trial but resourcing remained problematic due to assumptions of the efficiencies that would be realised by the platform design. Based on our practical experience, managing adaptions to the platform design, specifically the addition of a new treatment cohort, were resource intensive in the CTU and require similar resourcing as setting up a new trial. A flexible model would be preferable whereby a baseline resource is guaranteed with additional resource actively incorporated as the trial progresses to ensure adequate infrastructure is in place to support trial set-up activities whilst management of patient safety and data quality of ongoing cohorts continues in parallel.

### Central sample management and screening component considerations

Biomarker-driven platform designs, which provide the potential to screen for multiple treatment options within one trial, have been shown to be attractive to patients and to their clinicians [5, 8]. However, implementation can be burdensome for participating sites due to the inherent technical complexity and requirement for multidisciplinary engagement [1] especially related to sample collection and management. In plasmaMATCH, participating treatment sites were selected based on experience of delivering biomarker-guided trials and we conducted extensive face-to-face site initiation visits ensuring all personnel involved in the delivery of the trial (clinical, pharmacy, research nurses, trial coordinators and data managers) received appropriate protocol training. Enhanced guidance documentation including bespoke screening result reporting forms, patient pathway visual aids and comprehensive laboratory manuals and sample kits supported the protocol. Despite this, participating sites required significant ongoing support from the CTU trial team.

Central sample management was resource intensive in order to meet the ambitious turnaround times for screening results to be returned to participating sites for real-time clinical actionability. The turnaround time of results is a key practical issue that needs to be considered during the design phase of a platform trial. Estimated timelines should be realistic in terms of method of shipment to be used, the capabilities of the central laboratory and well communicated to the participating sites in order to manage expectations and the patient pathway appropriately. If the turnaround time is too long, investigators may be reluctant to delay patient treatment awaiting results and therefore recruitment can suffer as a result. The timing of the test is important too, test too early (e.g. before progressive disease or too early in progressive disease), and this may affect the validity of the result.

Whilst the plasmaMATCH trial recruited ahead of target, we found that successful recruitment was driven by a small number of highly experienced and motivated, large treatment sites with the research infrastructure to support local delivery of the trial. We emphasise the importance of perceived conversion rate amongst investigators participating in the trial in order to maintain motivation and enthusiasm for the trial at participating sites. Consideration should be given to the number of targeted mutations screened for and the mutation frequency both during the initial design stage and at planned adaptions so as to allow a reasonable chance for an actionable result prompting entry into a therapeutic cohort and continued interest in the trial which is important for recruitment.

### Methodology considerations

Platform designs have raised regulatory concerns regarding scientific value of reported outcomes and data integrity. These include assessment of multiple IMPs in small sub-groups of patients, with the potential for control of type I error associated with shared control groups and impact of closed cohorts on the conduct of ongoing cohorts. It is imperative that statistical design considerations have been appropriately addressed from the outset [4, 8, 17, 18].

From a statistical point of view, the plasmaMATCH phase II, non-comparative trial design, and therefore statistical analysis plan, was fairly straightforward. The parallel treatment cohorts were intentionally designed as phase II to provide proof-of-principle activity for the targeted therapies, analysed independently and therefore not requiring a control group. The primary endpoint was confirmed objective response and treatment continued until disease progression or unacceptable toxicity. Regardless of the regulatory requirements for a single end of study report, given the single CTA for plasmaMATCH, the intention from trial conception was to ensure that the primary analysis for each treatment cohort was performed and results presented as soon as available to ensure data transparency.

One of the key challenges associated with the platform design relates to appropriate safety oversight of multiple novel and unlicensed IMPs. In plasmaMATCH, regular quarterly IDMC meetings were held to review and advise on safety signals in the accumulating data. A separate safety review committee (SRC) was also convened to review accumulating cohort A data regularly due to the unlicensed increased dosing schedule used for the IMP. Close working relationships with the trial statisticians, data managers and a dedicated clinical research fellow were essential to ensure complete, high-quality data within a timely manner for the frequent SRC and IDMC review of the emerging safety data, given the fast recruitment rate. A project plan for data management processes in particular was vital in the lead up to the primary analysis of the screening component and treatment cohorts to ensure all tasks were identified, delegated appropriately and completed in time to meet deadlines for presentation.

## Conclusions

plasmaMATCH is an example of a successful platform trial which demonstrated ctDNA mutation analysis worked and that the designs of the treatment cohorts were able to identify treatment activity that will be explored further. plasmaMATCH demonstrates that platform trial designs can be efficient, allowing evaluation of multiple targeted therapies concurrently within one protocol and reporting of individual cohorts whilst others continue, provided that the operational challenges discussed herein and summarised in Table 1. Operational recommendations based on practical experience of the plasmaMATCH trial are recognised in advance and considered during trial development. Having an integrated screening component and detailed cohort-specific appendices within one protocol allows for efficient conversion from molecular target identification to treatment. However, the complexity and workload associated with managing multiple treatment cohorts and opening new cohorts whilst others are ongoing or reporting should not be underestimated or under resourced, nor should the importance of engaging with an established clinical trials infrastructure, such as a CTU, with experience of managing and analysing complex trial designs. Funding for these designs can prove difficult due to their inherent size and complexity despite relatively small patient numbers involved in individual treatment cohorts. An integrated funding model involving multiple partners can achieve the level of funding required for a platform trial supported by a mutual confidentiality agreement between partners and bespoke funding agreement to ensure consistency. Obtaining regulatory approval for platforms can be time consuming therefore early engagement during trial design is key. Whilst designs incorporating molecular screening and multiple treatment options are attractive to patients and their care teams, the associated logistical burden of managing multiple documentation and patient pathways locally can overwhelm participating sites. Site selection should be carefully planned during the development of the trial concept in communication with potential sites and additional resource allocated throughout the trial to allow for closer collaboration in support of the research teams at sites. The operational challenges can be overcome with sufficient resources and planning. Greater recognition of the scale and complexity of these designs can ensure appropriate resource and infrastructure is in place to support safe and efficient trial conduct.

## Data Availability

N/A.

## Abbreviations

CI: Chief investigator
CTA: Clinical trial application
ctDNA: Circulating tumour DNA
CTA: Clinical trials unit
eCRF: Electronic case report form
GP: General practitioner
ICF: Informed consent form
ICR: Institute of Cancer Research
ICR-CTSU: Institute of Cancer Research Clinical Trials and Statistics Unit
IDMC: Independent data monitoring committee
IMPs: Investigational medicinal products
NGS: Next generation sequencing
NIHR: National Institute for Health Research
PIS: Patient information sheet
RMH: Royal Marsden NHS Foundation Trust
SRC: Safety review committee
TNBC: Triple-negative breast cancer
